# Different Outcomes of Acute Encephalopathy after Status Epilepticus in Patients with Dravet Syndrome. How to Avoid Them? Comment on De Liso et al. Fatal Status Epilepticus in Dravet Syndrome. *Brain Sci.* 2020, *10*, 889

**DOI:** 10.3390/brainsci11060792

**Published:** 2021-06-16

**Authors:** Charlotte Dravet

**Affiliations:** Child Neuropsychiatry Unit, Fondazione Policlinico Universitario Agostino Gemelli, IRCCS, Università Cattolica del Sacro Cuore, 00168 Rome, Italy; charlotte.dravet@free.fr

Dear Editor

I read with interest the article “Fatal Status Epilepticus in Dravet Syndrome” by Paola De Liso et al. recently published in this journal [1]. The authors report in detail the cases of seven patients with Dravet syndrome who died after a febrile epileptic status (ES). They discuss the risk factors that can provoke this complication and the mechanisms that could explain the fatal issue, and ask for a therapeutic protocol in order to avoid this fatal issue. I would like underline that this type of complication, known as “Acute encephalopathy after febrile epileptic status” does not always provoke the death of the patient. That is well reported in other publications [2,3]. In particular, Tian et al. [4] reported 35 cases, including 12 patients who died and 23 who survived with massive, often irreversible, neurological regression. They note several predicting factors: early age at onset, epilepsy severity with strong pharmaco-resistance and repetitive ES, age under 5 years at the time of the event, and high fever. Here I give an example of this situation in one family. Two sisters suffering from Dravet syndrome (incomplete form without myoclonia) presented several ES during the course of their disease without complication and without neurological regression (Table 1).

However, the last ES, which occurred independently in the two patients at an interval of five months, progressed as an acute encephalopathy. The youngest died, whereas the oldest survived. The later had important neurological sequelæ, but she progressively recovered with an intensive rehabilitation; at 18 years she had recovered around 70% of her previous competences. The anamnesis of the two girls reveals that they presented some common points: severe epilepsy in the first years with repetitive ES, not a high seizure frequency before the status with the same antiepileptic drugs, and high fever during the status. However, they also presented differences: age at the epilepsy onset, age at the time of the event, coma duration, and different brain injury on MRI. The two girls carried the same *SCN1A* variant, inherited from the father. One explanation for the better outcome of Patient 2 could be her age, 15 years 9 months, and a low seizure frequency during late childhood and adolescence.

This type of ES is different from the usual ES which punctuate the course of Dravet syndrome. This acute encephalopathy is well known, but not explained, by the Japanese authors [5] who have shown the presence of a cerebral edema in MRI. In the cases reported by Myers et al. [6], the cerebral edema displayed by an early MRI led to a severe brain herniation that probably produced the death. The cause of this edema remains unclear, but the high temperature seems to be one prominent factor. Even if a viral infection could be the origin of this acute encephalopathy, we do not know why it is so severe. However, as advised by De Liso et al., we should alert the families and the doctors on its possible occurrence and the ways of managing it [7]. We know that these patients are prone to having “febrile” seizures. We also know these seizures are usually not provoked by a true fever (temperature higher than 38 °C), but by a temperature variation often lower than 38 °C. So, parents and caregivers should be worried when the temperature during a seizure is going up unusually in spite of the administration of antipyretic and antiepileptic drugs and they should go to the hospital quickly. If the situation persists in the emergency room, in addition to a continuous electroencephalogram, an MRI should be performed as soon as possible to detect abnormal images and signs of cerebral edema, then an antiedema treatment should be added and etiological investigations should be performed. Though the antiedema treatment did not avoid the fatal issue for the patients reported by Myers et al., it could be efficient if given at the very onset of the episode. In case it is not possible to have an MRI in an emergency, a CT scan could be performed. However, in the absence of imaging confirmation, the antiedema treatment should be implemented quickly.

## Figures and Tables

**Table 1:** Data of the two patients.

	Patient 1	Patient 2
Date of birth	15 September 2006	16 April 1999
Sex	Female	Female
Age 1st seizure	3 m 1/2	5 m 1/2
Cognitive development	Mild retardation, obsessive traits	Mild retardation
*SCN1A* variant	c.4973C>G p.Thr1658Arg in heterozygose, inherited from the father	c.4973C>G p.Thr1658Arg in heterozygose, inherited from the father
Other ES	Several febrile ES, one for 4 h, not followed by coma and regression	Several febrile ES in infancy and childhood, not followed by coma and regression
Seizure frequency during the year before this status	Less than monthly	No seizure in the last 22 months
Date of the event	6 June 2015	17 January 2015
Age at the event	8 y 9 m	15 y 9 m
Current treatment	VPA 1450 mg (40 mg/k/d→115 μg/mL)	VPA 675 mg (15 mg/k/d→90 μg/mL) CLB 10 mg
	CLB 10 mg (0.3 mg/k/d)	
	LEV 1800 mg (50 mg/k/d)	
First symptoms	Throat ache, mild fever, paracetamol, clonic seizure, IR DZ 10 mg (0.3 mg/k) at home, then vomiting, respiratory distress→hospital	1 seizure during sleep (unknown duration); no fever; oral MDZ 10 mg; T 39 °C; coma→hospital
At hospital status	Seizure duration 2 h 30	repeated seizures during 3 days, with coma and fever
temperature	38–40 °C	
Treatment	MDZ IV bolus (0.1 + 0.2 mg/k)	MDZ IV bolus (0. 1 mg/k × 3) followed by continuous IV MDZ
	LEV IV bolus (dose?)	
	PHT IV (15 mg/k)	antibiotics + antipyretics
	PB IV bolus (10 mg/k)→stop seizures→persistent coma	
MRI	**Day 5**: diffuse cortical swelling + hypersignal subcortical white matter	**Day 16 (after the end of the episode)**: enlarged ventricular cavities and subarachnoid spaces at the supra- and under-tentorial level, atrophy of the cerebellar hemispheres. Previously existing abnormalities.
	**Day 20**: diffuse cortical atrophy + central nuclei and subcortical white matter atrophy	
Outcome	**Persistence of coma**	**Coma duration: 3 days**
	Probably not epileptic tonic seizures	
	Treatment: oral VPA, LEV, CLB + IV MDZ	General regression with motor, cognitive, speech deficit, improved by intensive rehabilitation. 18 years: 75% recovery. No seizures. Treatment: VPA + CLB id
	**2 months later: severe sepsis and death.**	

Legend. ES: epileptic status; VPA: valproic acid; CLB: clobazam; LEV: levetiracetam; IR DZ: intrarectal diazepam; IV: intravenous; MDZ: midazolam; PHT: phenytoin; PB: phenobarbital; AEDs: antiepileptic drugs; MRI: magnetic resonance imaging.
